# Fall Classification by Machine Learning Using Mobile Phones

**DOI:** 10.1371/journal.pone.0036556

**Published:** 2012-05-07

**Authors:** Mark V. Albert, Konrad Kording, Megan Herrmann, Arun Jayaraman

**Affiliations:** 1 Sensory Motor Performance Program, Rehabilitation Institute of Chicago, Chicago, Illinois, United States of America; 2 Department of Physical Medicine and Rehabilitation, Northwestern University, Chicago, Illinois, United States of America; 3 Center for Bionic Medicine, Rehabilitation Institute of Chicago, Northwestern University, Chicago, Illinois, United States of America; 4 Max Nader Center for Rehabilitation Technologies and Outcomes Research, Rehabilitation Institute of Chicago, Northwestern University, Chicago, Illinois, United States of America; University Hospitals of Geneva, Switzerland

## Abstract

Fall prevention is a critical component of health care; falls are a common source of injury in the elderly and are associated with significant levels of mortality and morbidity. Automatically detecting falls can allow rapid response to potential emergencies; in addition, knowing the cause or manner of a fall can be beneficial for prevention studies or a more tailored emergency response. The purpose of this study is to demonstrate techniques to not only reliably detect a fall but also to automatically classify the type. We asked 15 subjects to simulate four different types of falls–left and right lateral, forward trips, and backward slips–while wearing mobile phones and previously validated, dedicated accelerometers. Nine subjects also wore the devices for ten days, to provide data for comparison with the simulated falls. We applied five machine learning classifiers to a large time-series feature set to detect falls. Support vector machines and regularized logistic regression were able to identify a fall with 98% accuracy and classify the type of fall with 99% accuracy. This work demonstrates how current machine learning approaches can simplify data collection for prevention in fall-related research as well as improve rapid response to potential injuries due to falls.

## Introduction

Falls in the elderly are a relatively common occurrence that can have dramatic health consequences. For people over 75 years old, the estimated incidence of falls is over 30 percent per year [1]. Nearly half of nursing home residents fall each year, with 40% falling more than once [2]. Falls can cause physical injury including fractures, head injuries, or serious lacerations. In community-dwelling patients who have fallen in a given year, the rate of serious injury is 5–10% [3], [4]. Falls can also elicit psychological consequences such as decreased independence [5] and increased fear of falling [6], [7]. This can lead to an avoidance of activity that can bring about a pattern of increasing isolation and deterioration [8], [9]. The impact of falls can be dramatic on certain populations, motivating the search for improved methods to minimize and respond to falls.

There are a number of strategies used to minimize falls [10]. There are physical training strategies such strength training [11] and balance training [12]. Modifying the home or workplace decreases the chance of falling [13]. The number of falls can be decreased by optimizing medication [14] or even having patients take nutritional supplements such as Vitamin D [15]. There are many therapies, medications, and lifestyle changes that can influence the probability of falling; however, at this time more research needs to be conducted. Improving the ability to track falls has the potential to streamline a wide range of fall-related research.

One objective and convenient way of documenting patient falls is through the use of mobile phones; most smart phones are equipped with accelerometers that can be used to detect when patients fall with exceptionally high accuracy. Falls are generally high-impact events, making detection simpler than identifying other daily activities. For example, some previous fall detection algorithms used only thresholds for the low freefall accelerations followed by high impact accelerations [16], [17]. Unfortunately, these studies either have very limited data sets (e.g. 3 young volunteers in very limited control circumstances) [16] or lower accuracy rates of nearly 80% for mobile phones [17]. Additional studies have simply observed the variations in a number of features [18]. Here we will apply a larger number of features, as has been done in previous studies using linear regression to predict falls [19], but use classifiers that have been successfully applied in activity recognition for mobile phones [20].

We use these classifiers to not only detect falls, but also to classify the particular type of fall. For example, in prosthetics research, adjusting aspects of a knee joint is often a tradeoff between which type of fall will occur. Different types of falls can result in different types of injuries. In the elderly a slip may be more or less likely to result in a serious injury than a trip simply because of the ease of breaking a forward versus backward fall. Also, because people tend to be more stable laterally, it is more likely when given a lateral fall that it may be from a loss of consciousness. Knowing the type of fall can be important for coordinating a more appropriate response to the fall, or adjusting the treatment to prevent a similar type of fall in the future.

The main focus of our study is to not only identify falls, but also classify their type. We can accomplish very high accuracy by using a much larger feature set than previous methods and by using current machine learning approaches to deal with the large number of resulting features. We demonstrate classification by having subjects simulate the different types of falls and record the resulting accelerations. For analyzing the ability to detect falls, these recordings are compared to fall-like events taken from a large dataset of everyday movements. Importantly, we show that our features and classifiers are capable of detecting and classifying the type of fall with a much higher level of accuracy than current techniques.

## Results

**Figure 1:**
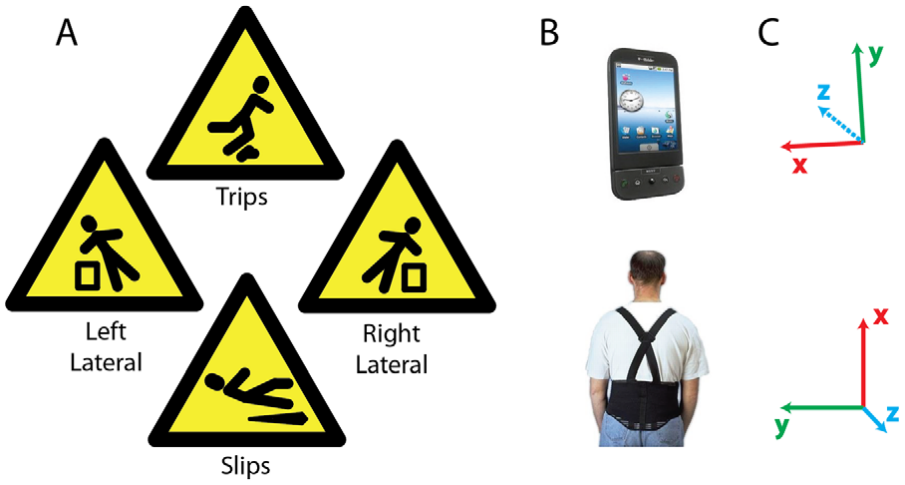
Types of falls measured, and axes of measurement. A) The four different types of simulated falls in this paper, positioned according to direction of the fall. B) The G1 android mobile phone that was used for recording, and the placement of the phone on the back of subjects. C) The axes of the tri-axis accelerometer relative to the images in B – xyz as red, green, blue, respectively. The phone was placed on the back of the subject so that the three axes pointed up, left, and to the back of the subject.

To create and validate the machine learning approaches for fall detection and classification, we collected two data sets. Fall data was obtained by simulating four different types of falls, representing four different directions in which someone could fall (slip-backward, trip-forward, left/right lateral – figure 1a) while subjects wore accelerometers on a belt, as detailed in the methods. Subjects also wore the accelerometers during everyday activities for a week, which measured a range of accelerations that could potentially be misclassified as false-positives. We applied five different machine learning classifiers to perform both the detection and the fall-type classification.

We collected the accelerometer data in a standardized way. Both a mobile phone and a separate, dedicated accelerometer were attached on belts and placed on the back of subjects. For simplicity, analysis is only shown and discussed for the mobile phone accelerometer. We found that the use of dedicated accelerometers provided comparable results to those presented for the mobile phone – although we note a possible ceiling effect as other classification strategies have shown significant improvements using dedicated accelerometers [17]. The orientation of the phone determined the orientation of the accelerometer axes. Because of the orientation of the phone on the belt, the x, y, and z-axes of the accelerometer corresponded to the up, left, and backward directions on the subjects (fig 1b and c). This setup was used to record from subjects for the four classes of simulated falls, as well as collecting recordings when the subjects wore the belts for a week. The goal of recording the data in this standardized way was to make interpretation clearer and analysis more accurate.

**Figure 2:**
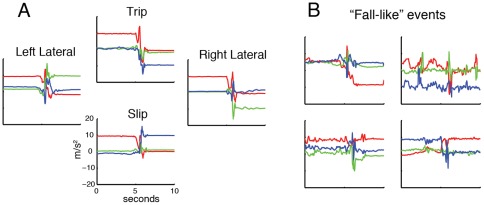
Example simulated falls and “fall-like” events. A) Falls of the four different types, arranged by direction of the fall as in fig 1a. B) Events that had relatively large changes in acceleration, and were extracted for comparison to simulated falls in the detection task.

We expected the different falls to have characteristic signatures in the recorded movements. For example, if someone falls forward, the z-axis would be oriented in the same direction as gravity at impact and likely after the fall. This regularity is evident in our displayed fall samples (fig 2a). However, not all falls could be classified that simplistically. Instead of searching for particular, directly observable features (e.g. high-impact accelerations), we chose to apply the standard, state-of-the-art machine learning approach: we constructed a large feature set (see methods) and had the algorithms select how to combine and weigh them appropriately.

We then needed data to evaluate our ability to detect falls. For this we selected samples of non-fall activities that were from everyday experience. Previous studies had subjects reenact activities of everyday living, such as sitting onto objects or walking [16], however, we decided to obtain a more natural and potentially more challenging control sample. By having the subjects wear the phones for a week, we collected a number of potential misclassification events. We selected the samples that were most fall-like based on the overall change in acceleration over a short, two-second interval. The selected average rate for these events was one per hour of recording. Although some samples appeared periodic in nature, many appeared similar to the simulated falls (fig 2b). Based on observations of these fall-like events, we believe these samples provided a better control group for fall detection than simulated daily activities.

**Figure 3:**
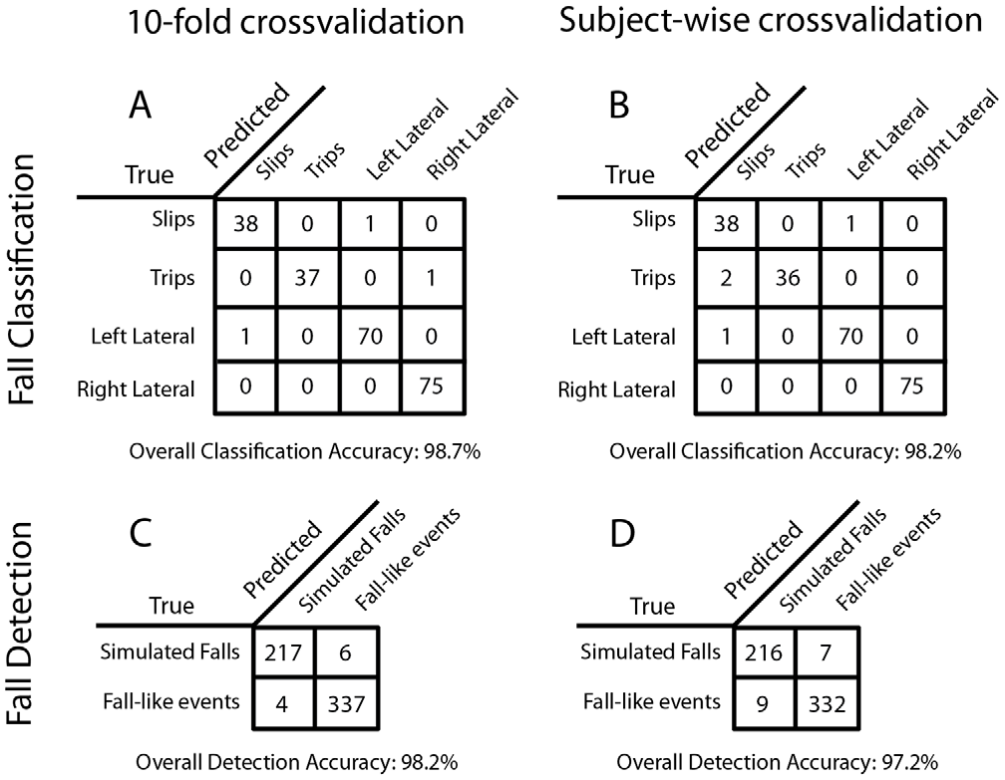
Crossvalidation results for detection and classification using Support Vector Machines (SVM's) on features extracted from the phone accelerometer recordings. Fall classification results when the task is to distinguish the type of simulated fall (A,B). Fall detection for distinguishing falls vs. high-impact “fall-like” events extracted from week-long recordings (C,D). Crossvalidation results are with all subject data pooled together (A,C) or subject-wise crossvalidated (B,D).

We first wanted to know how well the system could distinguish between the four different types of falls. We used five different classifiers: support vector machines (SVM), sparse multinomial logistic regression (SMLR, also referred to as regularized logistic regression), Naïve Bayes, k-nearest neighbors, and decision trees. We used the standard time series features as detailed in table 1. Using 10-fold cross-validation, SVM and SMLR classifiers were able to achieve 99% accuracy (SVM shown in figure 3a). To consider how well these classifiers would generalize to subjects for which there is no training data, we also performed subject-wise crossvalidation which only decreased the accuracy for SVM classification by 0.5% (figure 3b). In effect, both techniques had near perfect classification of the type of fall based on mobile phone accelerometer data.

**Table 1:** Features extracted from tri-axial accelerometer readings.

Values	Description	Total Features
moments	mean, absolute value of the mean, standard deviation, skew, kurtosis	15
moments of the difference between successive samples	mean, standard deviation, skew, kurtosis	12
smoothed root mean squares	no kernel, 5pt kernel, 10pt kernel	9
extremes	min, max, absolute value of the min and max	12
histogram	includes counts for +/− 4 z-score bins	27
Fourier components	32 samples from the Fourier spectrum	96
mean acceleration magnitude	e.g. 9.8 m/s^2^ if at rest	1
mean of the cross products	xy, xz, and yz	3
absolute value of the mean of the cross products	xy, xz, and yz	3

We also wanted to know how well the system could distinguish fall-like events in everyday life from actual, simulated falls. Importantly, the fall-like events were selected based on the highest-impact events from a set of eight week-long recordings. Both the SVM and SMLR classifiers achieved accuracies near 98% for pooled subject data when using 10-fold crossvalidation, while that accuracy decreased to only 97% when subject-wise crossvalidation was used. (fig 3c,d). In other words, across an average week of everyday movements there would be an estimated 2–3 non-falls that would be misclassified as falls. The combination of standard time-series feature sets and modern classifiers was able to detect falls with a high degree of accuracy.

**Figure 4:**
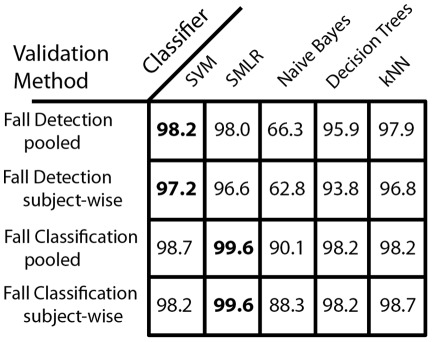
Classification accuracy for fall detection and classification of fall type for both pooled data using 10-fold crossvalidation and subject-wise crossvalidation. The most accurate classifier is indicated in bold.

We also wanted to demonstrate the efficacy of modern machine learning classifiers compared to more standard classifiers. Both SVM and SMLR are known to perform well with a large number of features. We wanted to contrast these with three more traditional classifiers (fig 4). Both decision trees and k-nearest neighbors performed at 94–98% accuracy for fall detection and 98–99% for fall type classification. Naïve Bayes performed poorly in both fall detection (63%–66%) and fall classification (88–90%) which can be expected because of the large number of potentially irrelevant features in our approach. Although all classifiers except Naïve Bayes report relatively high accuracies, SVM and SMLR classifiers are most accurate over all crossvalidation methods used, providing 98% accuracy for fall detection and 99% accuracy for fall classification.

## Discussion

We sought to use mobile phones and standard state-of-the-art machine learning to perform robust fall detection and classification. Instead of hand-picking the most relevant features, we used a large feature set, and had the relevant features selected by the algorithms. By applying classifiers such as SVM and SMLR, we showed that either popular technique performs well when large feature sets can be used, with near perfect accuracies in all cases. This was possible with the standard basic acceleration sensors found in almost all modern smartphones and could thus be implemented in phone apps.

Mobile phones are a convenient platform for recording movements, particularly measuring falls. They have built-in communication protocols that allow simple data logging to the device and wireless transmission. This permits real-time response or, in an experimental setting, compliance verification. Because mobile phones are widely adopted, compliance without verification is already high, as people are used to carrying them. Also, price is significantly reduced due to high production volume. Due to these advantages, mobile phones have the promise to provide a convenient, inexpensive, and objective means to track falls.

Previous work has shown how the accelerometers in mobile phones can be used to classify activities, including falls. Activities such as sitting, standing, walking, and running can be identified from mobile phone accelerations [21], [22], [23], [24], [25]. Our use of SVM's is also supported by work showing activity recognition and fall detection that can be over 95% accurate in both cases [20]; however, that work was done analyzing the movement of body position based on radio tags. Our experimental design is most similar to that of Lee and Carlisle [17] (e.g. simulated falls, phones and dedicated accelerometers) although their accuracy was approximately 80%. This was primarily a result of using a simple threshold-based classification strategy relying primarily on the maximum and minimum accelerations to detect a fall. By comparison, we appear to be getting a lot of mileage out of using these machine learning algorithms. Tolkiehn and colleagues [26] used a chest mounted accelerometer with a small feature set to obtain 84% detection accuracy. Uniquely, they also classified direction of fall with 94% accuracy. All of these previous fall detection and classification approaches either used smaller feature sets or simpler classification methods. We believe our work shows that the use of mobile phones for fall detection can be greatly improved when using modern machine learning strategies.

Although this study presents high accuracies, there are a number of issues that will still need to be addressed before such techniques could be used in a more applied setting. First, the mobile phones used here were placed in a standardized position. This allowed highly stereotypical measurements that aided accuracy ratings, but made the results less applicable to the way people carry their mobile phones every day – e.g. a smartphone in a pocket will certainly lead to lower accuracies due to the inconsistent ways it can be carried. Also, the fall-like events from the week-long recordings, though perhaps better than using certain simulated daily activities, may not have provided an adequate control data set. There is no guarantee that the fall-like events selected are a representative sample of potential misclassifications. This false-positive rate would have to be more directly assessed to consider viability for application. Although this result relies on standardized accelerometer positioning, and false positive rates for daily living were not fully explored, this does demonstrate a significant improvement in current fall detection and classification techniques.

Fall detection promises to be important in the context of healthcare. The impact on individuals has already been addressed in the introduction, but the injuries, psychological damage, and increased patterns of inactivity due to falls will also be an increasing burden to health care services [27]. Over time this burden is expected to increase dramatically; currently there are approximately 40 million people over age 65, and this number is expected to reach 86.7 million in 2050 [28]. Most dramatically, the number of people aged 85 or older, the age most likely to suffer health consequences from a fall, is expected to triple by 2050. For this reason, there is an increasing incentive for the health care industry to pursue methods to minimize the number of falls, decrease the type of falls that are more likely to cause injury, and improve emergency response when falls do occur. We believe that any work that facilitates research addressing this issue can impact populations prone to falls, but also the health care infrastructure tasked to care for them.

This work is motivated by the possibility of using the fall detection algorithms in real-world scenarios where patients only have to carry phones with the downloaded app installed. This possibility depends on alleviating two critical, practical concerns – maximizing battery life and minimizing the number of false positives. On T-mobile G1's running android version 1.6 we were able to record directly to memory for approximately 10 hours without recharging depending on the quality of the battery and amount of movement. This is impractical for normal, daily use. Systems such as iFall [29] and PerFallD [30] applied techniques such as variable sampling rates, background services, simplified processing, and minimizing power-intensive features like screen use and access to storage. With these adjustments, and by using newer phones, the apps were able to run in the background over the course of a day. An additional practical concern is how the application responds to a potential fall during the day. For example, after a fall, a person may not be able to respond but may still require medical attention. The previously mentioned applications [29], [30] were able to detect when a fall occurs and respond appropriately, however with those algorithms the rate of false-positives may have been a significant issue. If a potential fall is detected, you may want to ask the user to respond; if they don't respond you may send a message to personal emergency contacts; and if no action is taken by anyone, emergency first responders could be contacted. The number of times each of these interventions occurs should be minimized; otherwise the application may not only be impractical from the user's perspective, but also too costly. By applying the techniques used here, along with improvements in battery management, we believe that fall detection comes one step closer to improving the immediate medical responsiveness after individuals with disabilities fall.

The methods we applied for fall detection and classification are important tools for improving patient outcomes from fall-related research. It is clear that mobile phones can be used for fall detection based on the mobile phone accelerometer readings that were used to acquire the data here. In one sense, the feature selection is actually simpler in this paper because it is automated – so a large feature set can be applied. This advances the application of modern machine learning classifiers such as support vector machines and regularized logistic regression. The rich features sets and state of the art machine learning classifiers are simple and straightforward to use in practice. Based on the high accuracies reported here for both fall detection and classification, we believe that these tools should be considered when addressing the ability to automatically detect falls. Such improvements have the possibility to impact not only fall-related research studies, but may also someday enable practical emergency responsiveness for fall related trauma.

## Methods

15 Healthy subjects (8F/7M, ages 22–50) carried recording devices as they performed a series of simulated falls in the lab. Nine subjects also carried the devices for a week to record everyday behavior for later fall detection. Subjects wore a belt that held two accelerometers – one as part of an android mobile phone, and the other a special USB accelerometer. The phones were T-mobile G1 phones running the Android OS version 1.6. The sampling rate was variable between 15 and 25 Hz depending upon the amount of movement [31]. The dedicated USB accelerometers (Gulf Coast Data Concepts X6-2 Mini) sampled at a constant rate of 20 Hz. These accelerometers were attached together on a belt and centered in the back. The phone was positioned such that the accelerometer x, y, and z axes were directed up, left, and behind the subject, respectively (see fig 1). Written, informed consent was obtained for all subjects. The Northwestern University institutional review board specifically approved this study.

### Simulated falls and fall-like events

The subjects that performed simulated falls were instructed to perform four different classes of falls – slips, trips, left lateral, and right lateral falls. In all cases, subjects were asked to approximate the fall type and fell onto pads. The original design separated lateral falls into two types, active falls and faints. These two different types of falls were combined to simplify analysis. Each subject was instructed to perform each fall type three times for a total of 18 times per subject.

In order to record data for fall detection analysis, nine subjects also carried the accelerometers for one week. From these recordings, samples of “fall-like” events were extracted to compare to the data from simulated falls. Fall-like events were times in which the acceleration changed drastically in a short period of time. Due to battery use, only portions of the week were recorded. Specifically, the average squared change in acceleration was smoothed using a two second running average. 10 seconds clips were taken at the maximum values of this function. For every hour of recording, one sample was extracted.

### Data preprocessing and feature extraction

The accelerometer signals were preprocessed using the following procedure. The phone accelerometer values were linearly interpolated to match 20 Hz. All analysis was performed on 10 second clips of data centered on the falls, or fall-like events in the case of week-long recordings. From these 10 second clips, features were extracted, as summarized in table 1. This feature set was used for both classification tasks: detecting falls vs. non-falls and classifying the particular type of fall.

### Classification algorithm

Five different classification algorithms were used for detection and classification: support vector machines (SVM) [32], sparse multinomial logistic regression (SMLR) [33], naïve Bayes, decision trees, and k-nearest neighbors. Many of these techniques have been successfully applied in a large number of machine learning classification problems with a great deal of practical success on large feature sets. We normalized each feature to have mean 0 and unit variance. The SVM classifier was from the LIBSVM package [32]. We applied radial basis functions, giving us two hyperparameters – the soft slack variable, C, and the size of the Gaussian kernel, γ. The accuracy was highly robust to small changes in the hyperparameters, so reasonable choices were obtained by a grid search of 10^x^ where x is an integer between -5 and 5. The values which gave the highest 10-fold cross-validation accuracy are reported (C = 10 and γ = 0.1). The logistic regression classifier (SMLR) used regularization (hence, “sparse”) by including a penalization term on coefficient weights. In effect, regularization is an automatic way of performing feature selection, which helps avoid overfitting, and greatly improves crossvalidation accuracy. By changing the coefficient on the penalization term, λ, the system is biased to use more or fewer features. The parameter, λ, was found in the same way as the hyperparameters for SVM, giving λ = 0.0001). k-nearest neighbors only require a hyperparameter ‘k’ for the number of neighbors to average from. In this case, k = 3 was found to maximize the 10-fold crossvalidation accuracy. Naïve Bayes and decision trees had no hyperparameters. We used the standard MATLAB implementation for the last three classifiers (NaiveBayes, classregtree, knnclassify).
